# Supplementary material for: Complexome profiling of the Chlamydomonas psb28 mutant reveals TEF5 as an early PSII assembly factor
Source: Plant Cell. 2025 Mar 17;37(6):koaf055. doi: 10.1093/plcell/koaf055 (PMC12164590; doi:10.1093/plcell/koaf055)
Supplement: koaf055_Supplementary_Data [file koaf055_supplementary_data.zip › psb28_interactive.zip!/plots/intensity/qualityPlots/peptides/PLPA9_Cre02.g143667.t1.1.html]

### Cre02.g143667.t1.1

Trivial name: PLPA9  
SeqCoverage [median]: 22.20  
Mass: 42.30 kDa  
  
 

| Sequence | MissedCleavage | Mass [Da] | wtIntensity | muIntensity | Score |
| --- | --- | --- | --- | --- | --- |
| DAVAPLAGALAGSAR | 0 | 1338.73 | 0.00 | 1816.62 | 75.48 |
| DSDPAPSGGGR | 0 | 1014.44 | 399.68 | 428.71 | 85.73 |
| LEAPQLPEPLRPPK | 1 | 1583.90 | 1089.42 | 14494.99 | 61.38 |
| LVYSSGFNSGSLGGR | 0 | 1499.74 | 1117.72 | 11939.52 | 118.90 |
| RPGPPAAGFPATLGQVYQR | 1 | 1982.05 | 855.37 | 10269.14 | 89.31 |
| SASFDVTYLDSTGLR | 0 | 1630.78 | 395.01 | 1964.16 | 116.24 |
| VYLRDSDPAPSGGGR | 1 | 1545.75 | 0.00 | 3048.92 | 56.57 |
|  |  |  | 3857.2 | 43962.1 |  |
